# ^13^C Pathway Analysis for the Role of Formate in Electricity Generation by *Shewanella Oneidensis* MR-1 Using Lactate in Microbial Fuel Cells

**DOI:** 10.1038/srep20941

**Published:** 2016-02-12

**Authors:** Shuai Luo, Weihua Guo, Kenneth H. Nealson, Xueyang Feng, Zhen He

**Affiliations:** 1 Department of Civil and Environmental Engineering, Virginia Polytechnic Institute and State University, Blacksburg, VA 24061, USA; 2 Department of Biological Systems Engineering, Virginia Polytechnic Institute and State University, Blacksburg, VA 24061, USA; 3 Department of Earth Sciences, University of Southern California, Los Angeles, CA 90089, USA

## Abstract

Microbial fuel cell (MFC) is a promising technology for direct electricity generation from organics by microorganisms. The type of electron donors fed into MFCs affects the electrical performance, and mechanistic understanding of such effects is important to optimize the MFC performance. In this study, we used a model organism in MFCs, *Shewanella oneidensis* MR-1, and ^13^C pathway analysis to investigate the role of formate in electricity generation and the related microbial metabolism. Our results indicated a synergistic effect of formate and lactate on electricity generation, and extra formate addition on the original lactate resulted in more electrical output than using formate or lactate as a sole electron donor. Based on the ^13^C tracer analysis, we discovered decoupled cell growth and electricity generation in *S. oneidensis* MR-1 during co-utilization of lactate and formate (i.e., while the lactate was mainly metabolized to support the cell growth, the formate was oxidized to release electrons for higher electricity generation). To our best knowledge, this is the first time that ^13^C tracer analysis was applied to study microbial metabolism in MFCs and it was demonstrated to be a valuable tool to understand the metabolic pathways affected by electron donors in the selected electrochemically-active microorganisms.

Microbial fuel cells (MFCs) are an emerging technology to convert organics in wastewater to electrical energy^1^,^2^. Electrochemically active bacteria (EAB) serve as effective microbial catalysts to transfer electrons from substrates to solid electron acceptors (e.g., an anode electrode) under anaerobic conditions^3^,^4^,^5^,^6^. Electron transfer from EAB to the electrode is one of the most important steps in electricity generation by MFCs, and can be critically influenced by the type of the electron donors that will result in difference in the composition, structure, and metabolism of microorganisms on the anode electrode^7^,^8^,^9^. Therefore, understanding the impact of electron donors on electricity generation in MFCs is important to improve the efficiency of energy recovery^10^,^11^,^12^.

*Shewanellaceae* is a typical family including several model bacterial species such as *Shewanella oneidensis* MR-1 that can generate electricity in MFCs^9^,^13^,^14^. *S. oneidensis* MR-1 can transport electrons via intracellular metabolic reactions to complete extracellular electron transfer (EET) pathways^15^, or via multiple electron-transferring mechanisms including direct contact with the electron acceptor^16^, redox reactions of mediators (e.g., flavin) to transfer electrons^17^,^18^,^19^, and/or conductive nanowire extension to contact the electron acceptor^20^. Versatile electron transferring abilities allow *S. oneidensis* MR-1 to use multiple organics as electron donors (e.g., lactate, pyruvate, acetate, formate) to process dissimilatory reactions for reducing insoluble metals (e.g. Fe(III), Mn(IV)) and some inorganic ions (e.g., nitrate, nitrite) as electron acceptors^13^,^21^,^22^,^23^,^24^. Among those electron donors, lactate has been extensively studied. It was found that *S. oneidensis* MR-1 prefers to catabolize lactate because of a series of internally competent enzymatic reactions that utilize the electron donors to obtain energy for growth and electricity generation^15^,^25^,^26^. Under anaerobic conditions, such as the anaerobic anode of an MFC, lactate can be utilized to release electrons with the involvement of NADH, specific cytochromes, as well as various complexes, and it is further oxidized to acetyl-CoA, and CO_2_ or formate^14^,^15^,^27^. To assess electricity generation efficiency, Coulombic efficiency (CE) and Coulombic recovery (CR) are quantitative indicators to show the fraction of collected electrons through EET versus total ideal electron production^28^,^29^,^30^. It was reported that the lactate-fed MFC inoculated with *S. oneidensis* MR-1 achieved CE at the range of about 15–20%^16^,^31^; thus, there is still a lot of room to improve electricity generation efficiency.

In addition to lactate, formate is another potentially important electron donor to serve energy source for *S. oneidensis* MR-1^13^,^15^,^32^. *S. oneidensis* MR-1 has NAD^+^-dependent formate dehydrogenase (FDH) to catalyze the formate oxidation to CO_2_, and release electrons to complete EET pathway^14^. On the basis of EET started from lactate and pyruvate utilization, the catabolic metabolism of formate with the help of FDH can release and transfer more electrons to specific electron acceptors; such a mechanism is possibly helpful to improve CE/CR *of S. oneidensis* MR-1 in MFCs^14^,^27^. Thus, we hypothesized that extra formate addition on lactate utilization can boost the electrical output and improve CE/CR. However, formate is seldom studied for its effect on electricity generation of *S. oneidensis* MR-1 in MFCs.

This study aims to investigate the role of formate for the catabolic metabolism and electricity generation of *S. oneidensis* MR-1 in MFCs. Particularly, we applied ^13^C tracer experiment, which is a method widely used to facilitate the elucidation of metabolic rewiring in various non-model environmental microorganisms^33^,^34^,^35^. Normally, by feeding the ^13^C labeled substrate into a culture system, the carbon flow in the intracellular metabolism is traced to produce labeled metabolites (e.g., proteinogenic amino acids). By analyzing these metabolites using gas chromatography-mass spectrometry (GC-MS) and the following natural isotopic correction of the raw mass spectra, the isotopic labeling patterns (e.g., mass distribution vector) would be obtained and could be directly analyzed to reveal the metabolic rewiring of the target strains. Our previous study has applied the ^13^C tracer experiments to a well-designed anaerobic system for revealing the distinct intracellular metabolic behaviors between the biofilm and planktonic *S. oneidensis* MR-1 cells^36^. The ^13^C tracer experiment successfully revealed the cell metabolisms of *S. oneidensis* MR-1, and a conclusion was made that C1 metabolism such as formate oxidation is strongly related to the electron transferring to the extracellular environment. Therefore, in this study, ^13^C tracer experiment was applied to help us rigorously analyze the effects of formate on microbial metabolism of *S. oneidensis* MR-1 using lactate for electricity generation in an MFC. The results indicated, for the first time, that the addition of formate in MFCs might synergize with the lactate utilization to improve the electricity generation efficiency, which was achieved by a unique metabolism in *S. oneidensis* MR-1 to decouple cell growth from electricity generation during co-utilization of lactate and formate.

## Results & Discussion

### Combined supply of formate and lactate enhanced current generation

**Figure 1:**
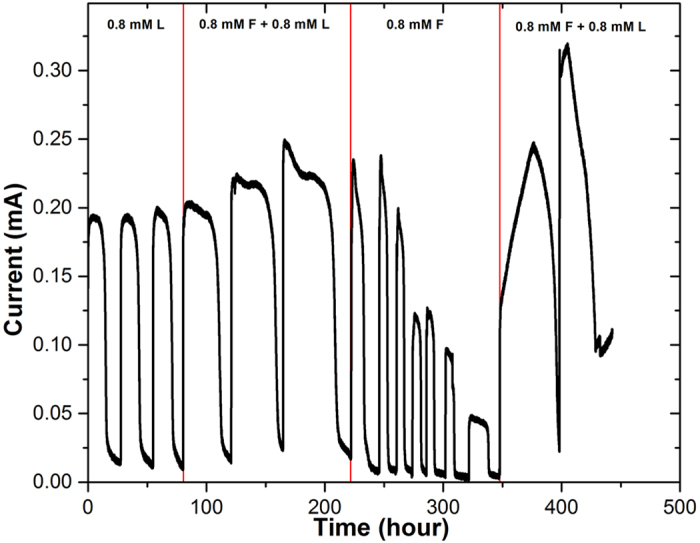
Current generation in the MFC supplied with various electron donors. Note: “L” means lactate; “F” means formate; “(F + L)” means addition of both substrate together; “0.8 mM” means 0.8 mM of each substrate added each cycle.

The MFC was operated under three conditions, each of which was for at least three repeated cycles and the current profile was recorded (Fig. 1). The added formate and lactate in the anode chamber was completely consumed in each cycle based on the HPLC results, indicating that the current drop at the end of each cycle was caused by the complete consumption of electron donors (Fig. 1). In the control reactor that did not have electricity generation (electrodes were disconnected under an open circuit condition), the electron donors were also consumed, possibly because of oxygen intrusion from the cathode (with continuous aeration) into the control chamber acting as the electron acceptor^37^. Though the oxygen diffusion through cation exchange membrane (CEM) was very limited^38^,^39^, the electron donor for each cycle could still be consumed because of its small amount (~0.8 mM) added into the control and anode chambers. Electricity generation in the MFC clearly competed for electron donors, and the comparable CR (Coulombic recovery) with other MFC studies of *S. oneidensis* MR-1 indicates that the anode electrode has strong ability to compete with oxygen for the electron donors to collect electrons (the data of CR were shown in the following section)^16^,^31^.

The peak current of the MFC was increased from 0.194 ± 0.005 mA using lactate alone to 0.231 ± 0.019 mA with additional formate as the extra electron donor (*p* < 0.05, one-tailed two-sample t-test with unequal variance at α = 0.05 for all the following statistical tests). Sole supply of formate significantly decreased the peak current to 0.147 ± 0.067 mA, lower than the current of other conditions (*p* < 0.05). When extra lactate was added with formate at the last two cycles, the current was boosted up again, to 0.290 ± 0.056 mA, significantly higher than the current production with sole formate supply (*p* < 0.05). The catholyte pH was stable at 7.2–7.4 due to generally low current output and strong buffering capacity of phosphate buffer saline (PBS)^40^, which eliminated the limiting effects of pH. The current generation with the combination of formate and lactate is higher than that with only lactate or formate, indicating that formate and lactate could have achieved synergistic effect when metabolized by *S. oneidensis* MR-1 to benefit the electricity generation in the MFC.

### Formate addition resulted in higher conversion efficiency

To further understand the impact of formate on the MFC performance containing *S. oneidensis* MR-1, CR (Coulombic recovery) and TC (total Coulombs) were used to reflect the conversion efficiency from electron donors to electrical output, with the equations below to calculate both parameters for each cycle (eqs 1, 2, 3)^28^,^29^,^30^:


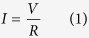



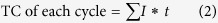



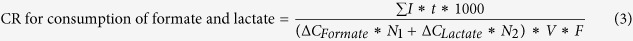


where in Eq. 1, *V* represents the voltage (V) and *R* is the external resistance (*R* = 8.2 Ω). In Eq. 2, *t* represents *t*he voltage measurement interval (t = 120 s) of the digital multimeter, and *I* represents the current (A) measured for each measurement interval with the assumption that the current is the same within a time interval. In Eq. 3, ∆*C*_*Formate*_ and ∆*C*_*Lactate*_ (mM) represent the moles of formate and lactate added into the reactor at the beginning of each cycle, respectively; N_1_ and N_2_ represent the mole of electrons transferring per mole substrate consumption, respectively, where N_1_ = 2 (formate → CO_2_ + 2H^+^ +2e^−^)^41^,^42^,^43^, and N_2_ = 4 (lactate + 2H_2_O → Acetate^−^+ HCO_3_^−^ + 5H^+^ + 4e^−^, incomplete oxidation in anaerobic condition)^16^,^31^,^44^,^45^; *V* represents the reactor volume (0.14 L); and *F* represents the Faraday constant (96485 Coulombs per mole electrons).

**Figure 2:**
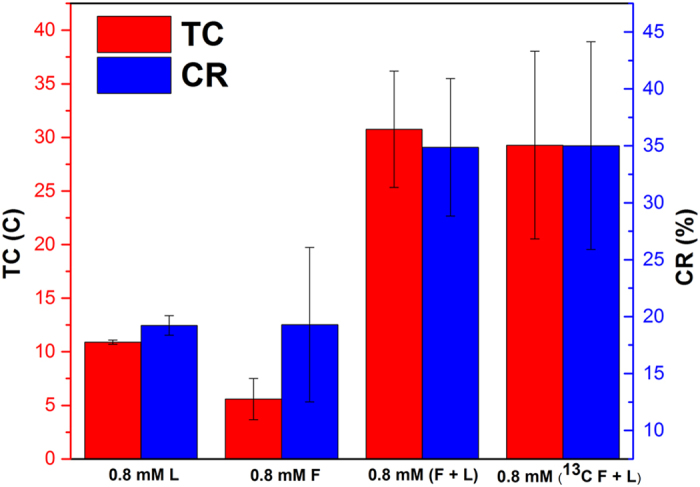
CR and TC obtained in the MFC under different supplies of formate and lactate. Note: “L” means lactate; “F” means formate; “(F + L)” means addition of both substrate together; “^13^C” represents the isotopomer addition; “0.8 mM” means 0.8 mM of each substrate added each cycle.

Combining non-labeled formate and lactate resulted in higher CR of 34.9 ± 6.0% than that of lactate alone (19.2 ± 0.9%) (*p* < 0.05), indicating that extra formate addition on lactate supply could enhance the efficiency of substrate conversion to electrons, which can be ultimately harvested by the solid electron acceptor in the anode chamber (Fig. 2). Supplying formate as the sole electron donor was found to have a lower CR of 19.3 ± 6.8% than that condition of combined formate and lactate (*p* < 0.05), suggesting that lactate was also needed for *S. oneidensis* MR-1 to achieve good electron transferring efficiency. The TC with formate addition to lactate was 30.8 ± 5.4 C, much higher than the sum of TC (16.5 ± 2.1 C) of both solely supplying formate (5.6 ± 1.9 C) and lactate (10.9 ± 0.2 C) (*p* < 0.05) (Fig. 2). These results demonstrate that the increased electricity generation with combined formate with lactate was not simply “1 + 1 = 2”; instead, the combination achieved “1 + 1 > 2”. This further confirms the pivotal role of formate in the electricity generation by *S. oneidensis* MR-1. Lactate is a commonly acknowledged as an electron donor in MFCs, being involved in the central metabolism of *S. oneidensis* MR-1^16^,^23^,^31^,^46^,^47^. Formate is more likely an electron transferring driving stimulus, which is related to the existence of FDH to convert the formate in periplasmic region to CO_2_ with the electrons being released extracellularly^14^,^27^,^48^. Therefore, to explain the synergistic effects of lactate and formate in electricity generation in MFCs, we hypothesize that lactate may be more involved in central metabolism while formate may be more related to EET.

### ^13^C tracer experiments facilitated the elucidation of the role for formate

**Figure 3:**
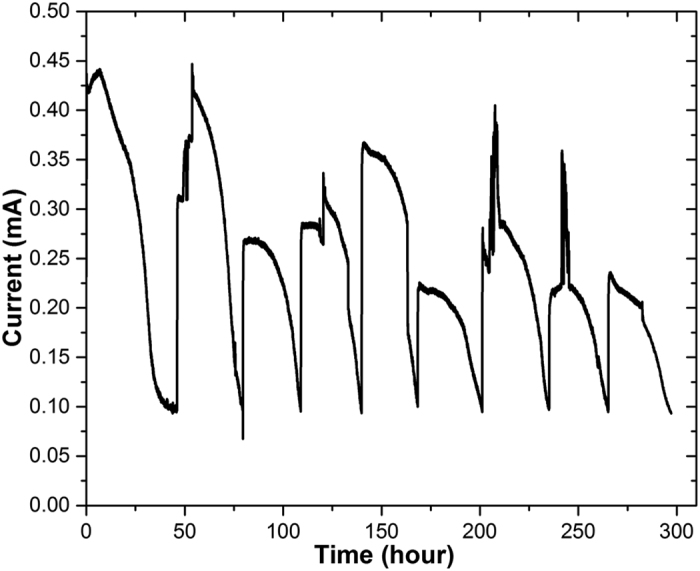
Current generation in the MFC supplied with 0.8 mM ^13^C isotopic formate and 0.8 mM non-labelled lactate.

To examine our hypothesis, we conducted the ^13^C tracer experiment to investigate the intracellular metabolism of *S. oneidensis* MR-1 when metabolizing formate and lactate in the MFC. The addition of ^13^C formate to non-labeled lactate also improved MFC electricity generation with the peak current of 0.308 ± 0.084 mA (Fig. 3), suggesting that the labeled formate was also favored by *S. oneidensis* MR-1 to produce electricity. When the labeled formate was used with non-label lactate, similar improvements in both CR and TC were obtained (Fig. 2). By examining the corrected mass distribution vectors from the isotopic analysis, no labeled carbon was found in the proteinogenic amino acids (Table 1), which are the building blocks for the cell growth of both planktonic and biofilm cells. This indicates that formate did not participate in the central metabolism to produce the building blocks for the cell growth. Instead, non-labeled lactate was mainly used for growth of the *S. oneidensis* MR-1. In other words, the cell growth and electricity generation might be decoupled when culturing *S. oneidensis* MR-1 with combined formate and lactate in MFCs. Indeed, it has been reported that lactate was a more preferential carbon substrate than formate for cell growth of *S. oneidensis* MR-1. The biomass yield of *S. oneidensis* MR-1 cultivating under an oxygen limited condition could reach 0.22 g/g when using lactate as the carbon substrate, much higher than 0.11 g/g with formate as the carbon substrate^49^. In addition, the expression levels of FDH genes in *S. oneidensis* MR-1 have been found to be up-regulated in MFCs^48^. Recently, a recombinant *S. oneidensis* MR-1 that harbored additional copies of FDH genes was found to generate a higher current density^14^, indicating the important role of formate in supplying electrons through FDH in MFCs. Considering the importance of lactate and formate for cell growth and electricity generation, respectively, it is interesting but unsurprising to find in this study that these two carbon substrates could synergize with each other to improve current generation via a decoupled metabolism of cell growth and electricity generation.

**Figure 4:**
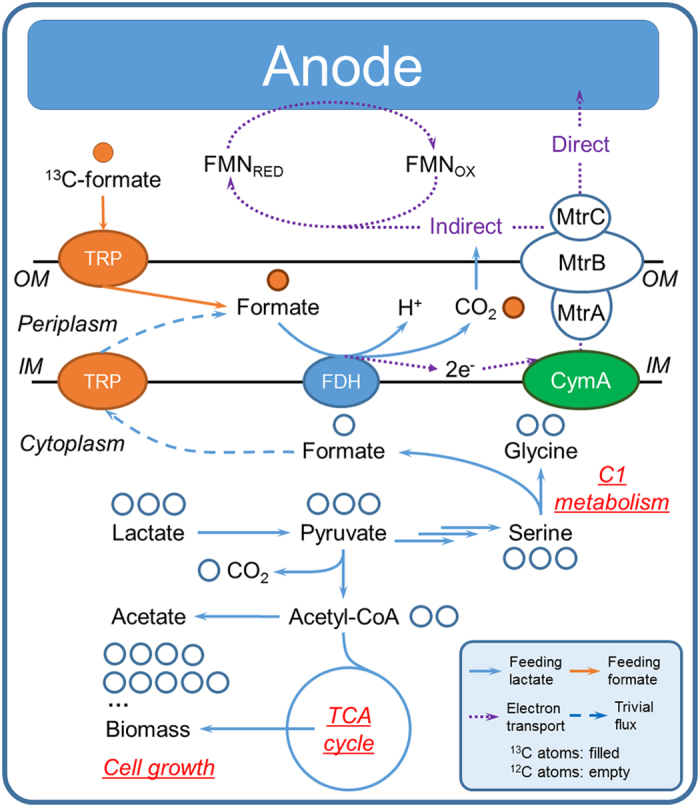
Proposed pathway of formate in the metabolism of *S. oneidensis* MR-1 after ^13^C formate experimental analysis. Designation: IM: inner membrane; OM: outer membrane; FDH: formate dehydrogenase; FMN_RED_: reduced flavin mononucleotide; FMN_OX_: oxidized flavin mononucleotide; CymA: tetraheme cytochrome anchored in inner membrane, accepts electrons from formate and transfers them to MtrA, MtrB and MtrC; MtrA, MtrB, MtrC: three kinds of periplasmic decaheme c-type cytochrome cytochromes anchored on outer membrane; TRP: formate transporter.

A metabolic pathway was proposed (Fig. 4), in which lactate is metabolized in the central metabolism of *S. oneidensis* MR-1 as its favorable substrate for cell growth^13^,^14^,^16^,^23^,^27^,^31^,^46^,^47^,^50^, while formate is more likely used as an electron-transfer driving stimulus from either extracellular exchange or the central metabolism to release electrons in periplasm by FDH^14^,^27^,^48^. In this scheme, the unlabeled lactate would be directly metabolized via central metabolism to produce various unlabeled building block (e.g., unlabeled amino acids) as we observed, and the labeled formate would be transported into periplasm and oxidized by inner membrane FDH to CO_2_ with the release of electrons, and eventually the labeled carbon was lost in the form of CO_2_ in the MFC. The released electrons from formate would be further transported by EET to the anode electrode or riboflavin intermediate to enhance electricity generation. Therefore, such decoupled cell growth from electricity generation could be responsible for the synergistic improvement of electricity generation with the addition of formate during the lactate uptake by *S. oneidensis* MR-1 in MFCs.

In summary, this study has contributed to an initial understanding of the role of formate in the electricity generation by *S. oneidensis* MR-1 using lactate in MFCs. The results show that the co-supply of two substrates would affect the EET behaviors, and ultimately the electricity generation in the MFC, implying the conception of synergy in substrates. Mutually complementary substrates may take advantage of substrate interaction in the cell metabolism, and generate a total effect greater than the sum of the individual contribution of single substrate for electricity generation. This may raise a question if other combinations of synergistic substrates also exist to enhance the MFC performance for pure or mixed culture. Moreover, ^13^C tracer experiment proves to be an effective technique to explore the influence of substrates on EET behavior of a selected species. Future studies can target what substrate has greater stimulating effect on EET behavior qualitatively and quantitatively to potentially improve the electricity generation. It will not only help reveal the pathway of EET and carbon flow, but also formulate a strategy for adjusting substrate combination to achieve optimal electricity generation under a certain special conditions (e.g., known composition of substrates being used for MFC application).

## Methods

### Bacterial strains and growing conditions in the MFC

*S. oneidensis* MR-1 (kindly provided by Dr. Y.J. Tang’s lab, Washington University, St. Louis, MO, USA) was initially grown in the shaking flasks with minimal medium containing 5 mM sodium L-lactate (Sigma-Aldrich, St. Louis, MO, USA) for two days at 100 rpm and 30 °C^24^,^46^. The culture medium was then transferred to the autoclaved anode chamber and the control chamber of the MFC system.

### MFC setup and operation

A three-chamber MFC was constructed by connecting three glass bottles together with two CEMs as separators (UltexCMI7000, Membranes International, Inc., GlenRock, NJ, USA) between each set of two bottles (Fig. S1). The liquid volume of three chambers was 140 mL/each. The middle chamber served as a cathode chamber, while one bottle containing an anode electrode connected to the cathode electrode was the functioning anode and the other bottle (also containing an anode electrode but under the open circuit condition) was the control chamber. The anode and control chambers were sealed with creamy epoxy to create oxygen-limited environment. The anode electrode was a piece of rectangular carbon cloth (2.5 cm × 4.5 cm PANEX^®^ 30PW06, Zoltek Corporation, St. Louis, MO, USA), and the cathode electrode using the same carbon cloth coated with platinum/carbon as the catalyst for oxygen reducing reaction (0.1 mg Pt cm^−2^). The cathode chamber was continually aerated to provide sufficient oxygen for cathodic reaction. The catholyte was 50 mM PBS (2.65 g L^−1 ^KH_2_PO_4_ and 5.35 g L^−1 ^K_2_HPO_4_), to buffer the catholyte pH.

The MFC was operated under a batch mode and at 30 °C with the external resistance of 8.2 Ω. Non-labelled electron donors were first supplied, and three conditions were tested: 1) 0.8 mM lactate in 1^st^–3^rd^ cycles; 2) 0.8 mM lactate +0.8 mM formate in 4^th^–6^th^ and 14^th^–15^th^ cycles; 3) 0.8 mM formate in 7^th^–13^th^ cycles. Once the current dropped to the baseline (~0.04 mA) in each cycle, the medium in the anode chamber and the control chamber was refreshed by removing 10 mL medium with sterile syringe and then injecting 10 mL fresh filtered (0.22 μm pore size) medium into the chambers. Depending on the substrate concentration, each cycle time period was 16–60 hours. To accelerate electron transfer, 0.2 μM riboflavin was added as an electron mediator to the anode and the control chambers at the beginning of the entire experiment (there was no more addition until the end of the experiment).

### ^13^C Tracer Experiment

Isotopically-labelled formate as an electron donor was supplied after finishing the cycles with non-isotopic labeled electron donors in the MFC. The combination of 0.8 mM [^13^C] sodium formate (99% purity, Cambridge Isotope Laboratory, USA) and 0.8 mM non-labeled sodium L-lactate was supplied to the anode and the control chambers at the beginning of a new cycle. After nine-cycle tests, both the liquid culture and carbon cloth in the anode and the control chambers were extracted to collect the planktonic and the biofilm cells, respectively, followed by the protocol developed previously for the isotopic analysis of proteiogenic amino acids^36^,^51^,^52^. In general, the biomass was hydrolyzed using 6 M HCl (20 h at 100 °C). The amino acids were derivatized in 50 μl of tetrahydrofuran and 50 μL of N-(tert-butyldimethylsilyl)-N-methyl-trifluoroacetamide (Sigma-Aldrich). A gas chromatograph (GC2010, Shimadzu) equipped with a SH-Rxi-5Sil column (Shimadzu) and a mass spectrometer (QP2010, Shimadzu) was used for analyzing the labeling profiles of metabolites. Three types of charged fragments were detected by GC-MS for Ala, Gly, Ser, Asp and Glu: the [M-57]^+^ group (containing unfragmented amino acids); and the [M-159]^+^ or [M-85]^+^ group (containing amino acids that had lost an α-carboxyl group). For each type of fragments, the labeling patterns were represented by M0, M1, M2, etc., which were fractions of non-labeled, singly labeled, and doubly labeled amino acids. The effects of natural isotopes on isotopic labeling patterns were corrected by previously reported algorithms^53^.

### Data Measurement

The voltage of the MFC was recorded by a digital multimeter (2700, Keithley Instruments, Inc., Cleveland, OH, USA) with measurement interval of 2 min. The concentrations of formate and lactate at the beginning and the end of each cycle were measured by high-performance liquid chromatorgraphy (HPLC) (Shimadzu, Columbia, MD, USA) equipped with an Aminex HPX-87H column (Bio-Rad, Hercules, CA, USA) and refractive index detector (RID, 10A, Shimadzu), with the following program: column temperature, 65 °C; mobile phase, 0.5 mM sulfuric acid solution; flow rate, 0.6 mL/min. The electrolyte pH was measured by using a pH meter (Oakton Instruments, Vernon Hills, IL, USA).

## Additional Information

**How to cite this article**: Luo, S. *et al*.^13^C Pathway Analysis for the Role of Formate in Electricity Generation by *Shewanella Oneidensis* MR-1 Using Lactate in Microbial Fuel Cells. *Sci. Rep*. **6**, 20941; doi: 10.1038/srep20941 (2016).

## Supplementary Material

Supplementary Information

## Figures and Tables

**Table 1:** Mass distribution vectors of the key proteiogenic amino acids.

Cell sources	Anode-biofilm		Anode-planktonic		Control-biofilm		Control-planktonic	
Fragments	M-57	M-159	M-57	M-159	M-57	M-159	M-57	M-159
**Ala**								
M0	0.93	0.95	0.96	0.95	0.95	0.95	0.94	0.94
M1	0.06	0.03	0.04	0.02	0.05	0.03	0.05	0.03
M2	0.01	0.03	0.00	0.03	0.00	0.03	0.00	0.03
M3	0.00		0.00		0.00		0.01	
**Gly**								
M0	1.00	1.00	0.98	0.98	0.95	1.00	0.95	0.97
M1	0.00	0.00	0.02	0.02	0.05	0.00	0.05	0.03
M2	0.00		0.00		0.00		0.00	
**Ser**								
M0	0.98	0.99	0.97	0.98	0.88	0.94	0.93	0.96
M1	0.04	0.02	0.03	0.02	0.17	0.06	0.06	0.04
M2	0.00	0.00	0.00	0.00	0.00	0.00	0.01	0.00
M3	0.00		0.00		0.00		0.01	
**Asp**								
M0	0.96	0.94	0.92	0.94	0.84	0.91	0.88	0.93
M1	0.04	0.05	0.09	0.04	0.16	0.05	0.10	0.06
M2	0.00	0.01	0.00	0.00	0.00	0.02	0.03	0.00
M3	0.00	0.01	0.00	0.01	0.00	0.01	0.00	0.01
M4	0.00		0.00		0.00		0.00	
**Glu**								
M0	0.94	0.98	0.96	0.95	0.92	0.96	0.92	0.89
M1	0.06	0.01	0.03	0.05	0.10	0.03	0.08	0.10
M2	0.00	0.01	0.00	0.00	0.00	0.00	0.00	0.00
M3	0.00	0.00	0.01	0.00	0.01	0.00	0.00	0.00
M4	0.00	0.00	0.00	0.00	0.00	0.00	0.01	0.00
M5	0.00		0.00		0.00		0.00	
