# Supplementary material for: 13C Pathway Analysis for the Role of Formate in Electricity Generation by Shewanella Oneidensis MR-1 Using Lactate in Microbial Fuel Cells
Source: Sci Rep. 2016 Feb 12;6:20941. doi: 10.1038/srep20941 (PMC4751489; doi:10.1038/srep20941)
Supplement: Supplementary Information [file srep20941-s1.pdf]

Supplemental Information

^{13}C Pathway Analysis for the Role of Formate in Electricity Generation by *Shewanella Oneidensis* MR-1 Using Lactate in Microbial Fuel Cells

Shuai Luo[1#], Weihua Guo[2#], Kenneth Nealson[3], Xueyang Feng[2*], and Zhen He[1*]

[1] *Department of Civil and Environmental Engineering, Virginia Polytechnic Institute and State University, Blacksburg, VA 24061, USA*

[2] *Department of Biological System Engineering, Virginia Polytechnic Institute and State University, Blacksburg, VA 24061, USA*

[3] *Department of Earth Sciences, University of Southern California, Los Angeles, CA 90089, USA.*

[#] Those authors contributed equally to this work

* Correspondence and requests for materials should be addressed to X.F. (xueyang@vt.edu) or Z.H. (zhenhe@vt.edu)

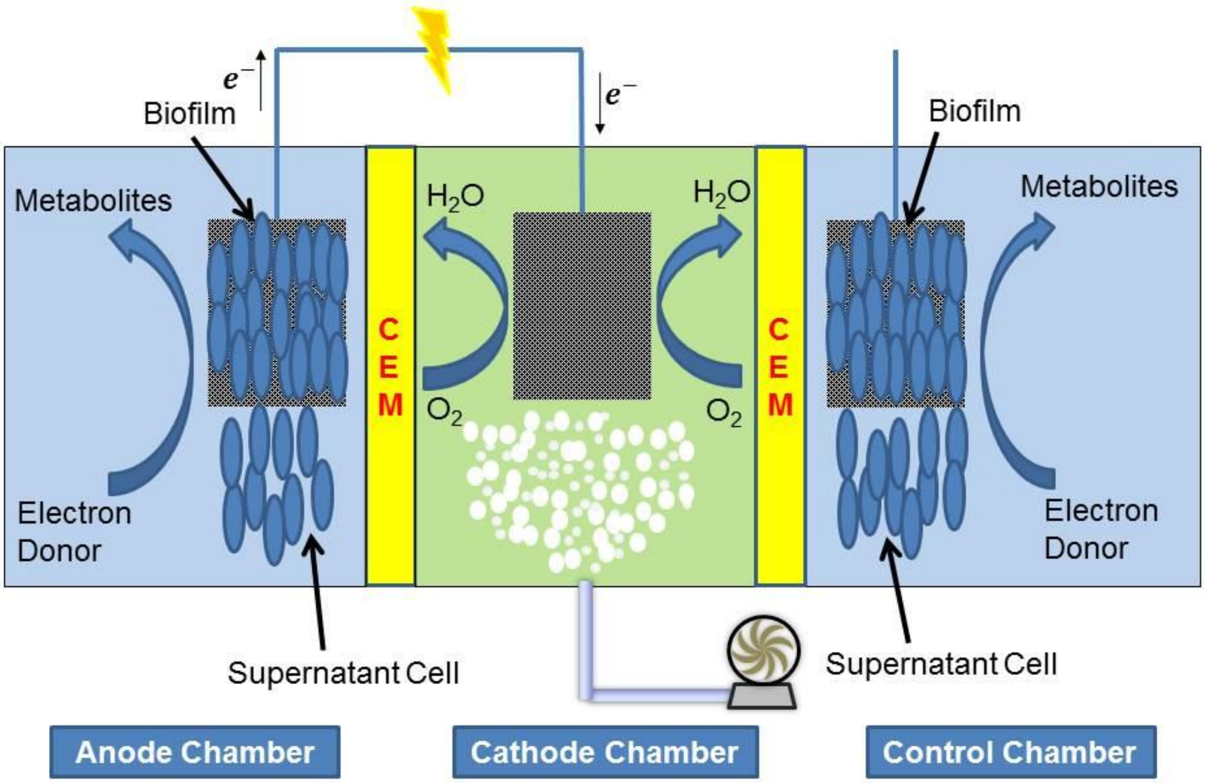

Figure S1. Schematic of a three-chamber system consisting of an anode chamber, a cathode chamber, and a control chamber. The electrodes in the anode and the cathode chambers were connected to form an electrical circuit (functioning MFC).
